# E-cigarette, or Vaping, Product Use–Associated Lung Injury Among Clusters of Patients Reporting Shared Product Use — Wisconsin, 2019

**DOI:** 10.15585/mmwr.mm6909a4

**Published:** 2020-03-06

**Authors:** Ian W. Pray, Sukhshant K. Atti, Carrie Tomasallo, Jonathan G. Meiman

**Affiliations:** ^1^Wisconsin Department of Health Services; ^2^Epidemic Intelligence Service, CDC; ^3^School of Medicine, Emory University, Atlanta, Georgia; ^4^Agency for Toxic Substances and Disease Registry, U.S. Department of Health and Human Services.

On July 10, 2019, Wisconsin Department of Health Services (WDHS) was notified of five previously healthy adolescents with severe lung injuries who reported use of e-cigarette, or vaping, products before symptom onset. As of December 31, 2019, 105 confirmed or probable cases of e-cigarette, or vaping, product use–associated lung injury (EVALI)* had been reported to WDHS . Three social clusters (A, B, and C), comprising eight EVALI patients (cluster A = two patients, cluster B = three, and cluster C = three) were identified. WDHS investigated these clusters with standard and follow-up interviews; laboratory analysis of e-cigarette, or vaping, products; and analysis of bronchoalveolar lavage (BAL) fluid. All eight patients reported daily use of tetrahydrocannabinol (THC)-containing e-cigarette, or vaping, product cartridges (THC cartridges) in the month preceding symptom onset. All THC cartridges were purchased from local illicit dealers, and all patients reported using THC cartridges labeled as “Dank Vapes,” among other illicit brand names. At least two members of each cluster reported frequent sharing of THC cartridges before symptom onset. All eight patients also reported daily use of nicotine-containing e-cigarette, or vaping, products. Vitamin E acetate (VEA) was detected in all five THC cartridges tested from two patients, and in BAL fluid from two other patients. These findings suggest that THC cartridges containing VEA and sold on the illicit market were likely responsible for these small clusters of EVALI. Based on information presented in this and previous reports (*1*,*2*) CDC recommends not using THC-containing e-cigarette, or vaping, products, especially those obtained from informal sources such as friends, family, or in-person or online dealers (*1*). VEA is strongly linked to the EVALI outbreak and should not be added to e-cigarette, or vaping, products (*1*).

A cluster was defined as two or more patients with confirmed or probable EVALI who directly shared e-cigarette, or vaping, products; obtained products from the same source; or reported a social connection and use of the same e-cigarette, or vaping, product brand names in the 3 months preceding symptom onset. All patients were interviewed by telephone using a standard EVALI questionnaire developed by WDHS, and five of the eight cluster-associated patients (A = one, B = three, C = one) completed in-depth follow-up telephone interviews to provide additional product use details. This included, for each product used, the dates of initiation and cessation, frequency and amount used, and the extent of sharing with other EVALI patients. In addition to interviews, one patient in cluster A and two patients in cluster C submitted a total of 11 e-cigarette, or vaping, products that were tested for the presence of VEA and other additives^†^ by the Food and Drug Administration (FDA), and BAL fluid from two patients (one each from cluster B and cluster C) were analyzed by CDC (*3*).^§^

Symptom onset for these eight patients ranged from June 18 through July 21, 2019 (Figure 1). Patients were aged 16–20 years (median = 17 years), and six were male. All eight patients reported daily use of THC cartridges purchased from local illicit dealers in the month before symptom onset. This included use of the Dank Vapes brand by all patients and an average of 2.6 unique brands of illicit THC cartridges per patient (range = one to five brands) (Table). At least two patients in each cluster reported frequent sharing of THC cartridges in the month preceding symptom onset, including concurrent use of the same cartridge in the same device (Table). On average, patients reported inhaling approximately one half of a 1-g THC cartridge daily (range = 0.2–1 cartridge per day) in the month before symptom onset; two patients (one in cluster B and one in cluster C) reported that this was more than usual for them. All patients also reported daily use of nicotine-containing e-cigarette, or vaping, products. These included commercial pods and refillable e-liquids purchased from retail locations or online. The amount of nicotine product use per day was not quantifiable because of variability among brands. Patients reported initiating use of THC cartridges a median of 9 months before onset of symptoms (range = <1 to 12 months) (Figure 2). Patients in cluster A initiated daily use of Dank Vapes 2–4 weeks before symptom onset, whereas patients in clusters B and C reported a longer duration of THC cartridge use before symptom onset, without changing brands or sources. All patients reported long-term use of nicotine-containing products, which were initiated a median of 33 months before symptom onset (range = 5–60 months) (Figure 2).

**FIGURE 1:**
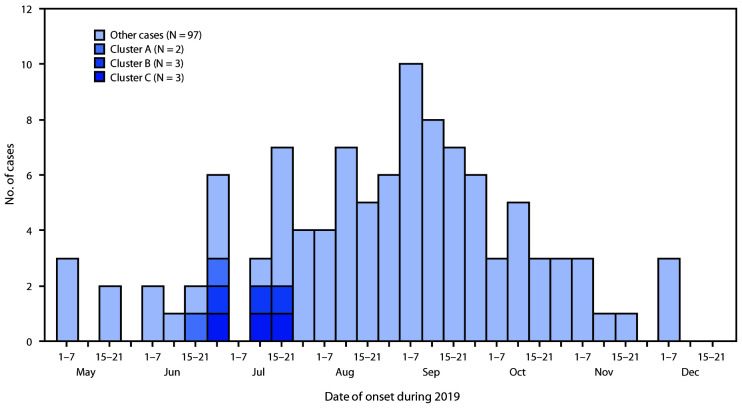
Dates of illness onset among 105 confirmed or probable e-cigarette, or vaping, product use–associated lung injury patients, including social clusters — Wisconsin, 2019

**TABLE:** Product use and clinical details for eight cluster-associated patients with e-cigarette, or vaping, product use–associated lung injury (EVALI) — Wisconsin, 2019

Patient no.	Cluster A		Cluster B			Cluster C		
	1	2	3	4	5	6	7	8
**Interview type**	Standard	In-depth	In-depth	In-depth	In-depth	In-depth	Standard	Standard
**THC product**								
Brand names	Dank Vapes	Dank Vapes	Dank Vapes	Dank Vapes	Dank Vapes	Dank Vapes	Dank Vapes	Dank Vapes
			Cookies	Cookies	Cookies	Chronic Carts	Chronic Carts	Cookies
			Dr. Zodiak			TKO	Supreme	Dr. Zodiak
			Mario Carts				Off-White	
							Monopoly	
Dose*	0.5	0.5	1	0.5	1	0.5	0.5	0.2
Months of use^†^	<1	<1	9	3	12	9	12	9
Product testing for VEA^§^	Not available for testing	2 of 2	Not available for testing	Not available for testing	Not available for testing	Not available for testing	Not available for testing	3 of 3
Social link and shared product use	Shared use^¶^		Shared use^¶^		Friend	Shared use^¶^		Friend,
	Same illicit dealer		Same illicit dealer		Same illicit dealer	Same illicit dealer		Unknown source
**Nicotine product**								
Brand names	N/A	Solace	Juul	Juul	Juul	Juul	Juul	Juul
		Nord	Salt-E			Jewel	Jewel	Vuse Alto
						Suorin	Air Factory	
Frequency	Daily	Daily	Daily	Daily	Daily	Daily	Daily	Daily
Months of use^†^	60	60	21	5	36	30	36	18
Product testing for VEA^§^	Not available for testing	Not available for testing	Not available for testing	Not available for testing	Not available for testing	Not available for testing	0 of 1	1 of 5**
**Clinical course**								
Hospital stay (days)	0	0	6	7	20	6	8	9
ICU	No	No	No	Yes	Yes	Yes	Yes	Yes
Intubated	No	No	No	No	Yes	Yes	No	No
BAL fluid testing for VEA	Not available for testing	Not available for testing	Positive	Not available for testing	Not available for testing	Not available for testing	Positive	Not available for testing

**Abbreviations:** BAL = bronchoalveolar lavage; ICU = intensive care unit; N/A = not available; THC = tetrahydrocannabinol; VEA = vitamin E acetate.

* Number of 1-g THC cartridges used per day in the month before symptom onset.

^†^ Number of months between reported initiation of product use and onset of EVALI.

^§^ VEA detected (number of products containing VEA of the total number tested). https://www.fda.gov/news-events/public-health-focus/lung-injuries-associated-use-vaping-products.

^¶^ Shared use is defined as directly sharing the same THC cartridges at the same time and place.

** One product was packaged as a THC cartridge but contained nicotine, VEA, and cannabinol.

**FIGURE 2:**
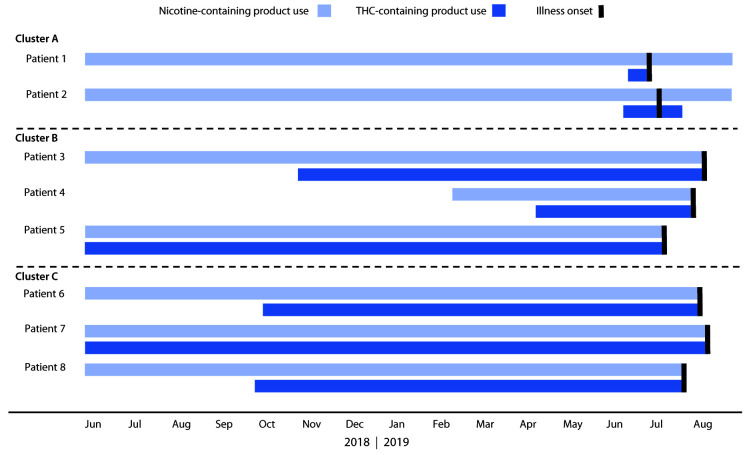
Dates of initiation* and cessation of nicotine- and tetrahydrocannabinol (THC)-containing product use and illness onset among eight cluster-associated e-cigarette, or vaping, product use–associated lung injury patients^†^ — Wisconsin, 2019 * All patients reported long-term use of nicotine-containing products, which were initiated a median of 33 months before symptom onset (range = 5–60 months). ^†^ The following is a summary of pertinent events for patients in Cluster C; similar patterns of product use initiation, sharing, and symptom onset were observed for patients in clusters A and B. Within cluster C, patient 6 and patient 7 were close friends who reported frequent sharing of Dank Vapes, Chronic Carts, and various other illicit THC cartridges before symptom onset, which occurred for both patients in early July 2019. All of the THC cartridges used by patients 6 and 7 were obtained from the same local illicit dealer, from whom they had purchased similar THC cartridges for the past 9–12 months. In the week preceding symptom onset, they reported using more than the usual quantity together, approximately one half of a 1-g cartridge per person per day; they also reported daily use of nicotine-containing e-cigarette, or vaping, products. Patients 6 and 7 developed nausea, vomiting, fever, and respiratory symptoms within 5 days of each other and stopped using e-cigarette, or vaping, products shortly after symptom onset. Patient 8 was a friend of patients 6 and 7 but did not report sharing products with them and was unsure if they shared the same local illicit dealer. This patient also reported daily use of Dank Vapes, among other brands, beginning 9 months before symptom onset, which occurred 2 weeks before that of patient 6. All three patients were hospitalized in the intensive care unit, and one required mechanical ventilation. Bronchoalveolar lavage fluid from patient 6 tested positive for vitamin E acetate, and all three THC cartridges from patient 8 contained vitamin E acetate.

Eleven e-cigarette, or vaping, products from three patients were tested. All five THC cartridges collected from two patients contained VEA; one product contained nicotine, VEA, and cannabinol; none of the five commercial nicotine products collected from two patients contained VEA. None of the products tested contained significant levels of other toxicants included in the FDA testing protocol. BAL fluids were tested for two patients, and both contained VEA; no other potential toxicants were identified in these BAL fluids.

Injury severity and clinical course varied among these eight patients (Table). Six patients were hospitalized for a median of 6.5 days (range = 6–20), five were admitted to the intensive care unit, and two required mechanical ventilation. Two patients from cluster A received a diagnosis of EVALI in outpatient settings. One patient from cluster B reported persistent respiratory symptoms 3 months after discharge.

## Discussion

Consistent with previous reports (*1*,*2*), THC cartridges containing VEA were closely linked to these small EVALI clusters. Nationwide, 80% of hospitalized EVALI patients reported use of THC-containing e-cigarette, or vaping, products, and 56% of EVALI patients with data on product use specifically reported using Dank Vapes in the 3 months preceding symptom onset (*4*). Similar results have been reported in Illinois, Wisconsin (*5*), and Utah (*6*), which, together, suggest that Dank Vapes and other illicit THC-containing products obtained from informal sources played a major role in the nationwide EVALI outbreak. The current findings reinforce this relationship by linking multiple EVALI patients to the same illicit THC cartridges. Although the specific sources of shared THC cartridges were not provided by patients, law enforcement activity in Wisconsin during that time indicates that counterfeit THC cartridges were being packaged and sold under the same brand names as those shared by EVALI patients, and could represent a potential source.^¶^ VEA was detected in THC cartridges or BAL fluids from at least one patient in each cluster, suggesting that the presence of VEA in illicit THC cartridges likely played a role in these clusters as well. This is consistent with the detection of VEA in BAL fluids from 48 EVALI patients in 16 states (*2*), and THC cartridges obtained from patients nationwide (*6*,*7*) and law enforcement in Minnesota (*8*).

The duration of THC cartridge use before symptom onset among these patients is an important new insight of this report. Patients began using THC cartridges a median of 9 months before illness onset, but this ranged from <1 month among patients in cluster A to 12 months among some patients in clusters B and C. None of the patients reported any change in brand name or source over that period, yet all reported symptom onset within a similar window of time. This suggests that a change might have occurred in the constituents of illicit THC-containing e-cigarette, or vaping, products, including the addition of VEA, shortly before June 2019, when these patients began to have symptoms. This timeline is consistent with the spike in EVALI-related emergency department visits observed nationwide in June 2019 (*9*), and with law enforcement seizures in Minnesota that found VEA in all THC cartridges seized in a September 2019 raid, but not in any products seized in 2018 (*8*).

Frequent use of THC cartridges was notable among these patients. Seven of eight patients reported using at least one half of a 1-g THC cartridge per day before symptom onset. Patients estimated that a full 1-g THC cartridge corresponded to approximately 300 to 500 hits (i.e., inhalations) and would require nearly continuous use throughout a day to expend. Using THC-containing e-cigarette, or vaping, products more than five times per day was found to be significantly associated with EVALI in a case-control study of Illinois patients (*10*) and might be a contributing factor in the EVALI outbreak.

All cluster-associated patients reported daily use of nicotine-containing products. However, no patients reported exclusive use of nicotine-containing products, and all reported long-term use with no change in brands or patterns of use preceding symptom onset. Also, VEA was not detected in any of the five commercial nicotine products tested, suggesting that nicotine-containing products were not associated with EVALI among these eight patients.

The findings in this report are subject to at least four limitations. First, this analysis was restricted to a small cluster of EVALI cases in Wisconsin and might not be representative of the nationwide EVALI outbreak. Second, the majority of data for this report were collected in October 2019, approximately 4 months after initial symptom onset for most patients, and recollections of brand names, frequency, and initiation of product use are subject to recall bias. Third, testing of THC cartridges or BAL fluids for VEA was only possible for four of the eight patients, which limited the ability to draw a definitive linkage to VEA for all cases. Finally, only five of eight cluster-associated patients were reached for in-depth interviews, which limited the ability to assess shared product use among three patients not reached for follow-up.

These findings reinforce current recommendations to not use THC-containing e-cigarette, or vaping products, especially those obtained from informal sources (*1*). Moreover, vitamin E acetate should not be added to e-cigarette, or vaping, products. Adults using e-cigarette, or vaping, products as an alternative to cigarettes should not go back to smoking. Irrespective of the ongoing investigation, e-cigarette, or vaping, products should never be used by youths, young adults, or pregnant women (*1*).

**Summary**

**What is already known about this topic?**

E**-**cigarette, or vaping, product use–associated lung injury (EVALI) has been linked to the use of tetrahydrocannabinol (THC)-containing products and vitamin E acetate.

**What is added by this report?**

Three small patient clusters in Wisconsin reported frequent, shared use of THC cartridges obtained from informal sources before symptom onset. Vitamin E acetate was detected in all five THC cartridges used by two of the patients and in bronchoalveolar lavage fluid from two other patients.

**What are the implications for public health practice?**

These findings support the link between vitamin E acetate and THC-containing products obtained from informal sources in EVALI cases. CDC recommends that persons not use THC-containing e-cigarette, or vaping, products, particularly from informal sources.

## Footnotes

* https://www.cdc.gov/tobacco/basic_information/e-cigarettes/assets/2019-Lung-Injury-Surveillance-Case-Definition-508.pdf.

† FDA testing of 11 e-cigarette, or vaping, products included nontargeted testing with Fourier Transform Infrared Spectroscopy (10 products); gas chromatography mass spectrometry (GC-MS) (seven); and headspace GC-MS (three) to detect the presence of VEA, THC, and other compounds available in four chemical libraries (Aldrich Condensed Phase Library, High Resolution Nicolet Sampler Library, Wiley/ National Institute of Standards and Technology Library, and Designer Drug Library). Targeted testing included high-performance liquid chromatography with ultraviolet detection for nicotine (one) and cannabinoids (three); high-pressure liquid chromatography-ultraviolet, inductively coupled plasma mass spectrometry for heavy metals, liquid chromatography with mass spectrometry detection for synthetic cannabinoids, opioids, poisons, pesticides, and other toxins (three); fatty acid methyl ester GC-MS (two); and gas chromatography with flame ionization detection to quantify the amount of vitamin E acetate present in the sample (two).

§ Analysis of BAL fluids by CDC used isotope dilution mass spectrometry methods to evaluate the presence of specific toxicants of concern: vitamin E acetate, medium chain triglyceride oil, plant oils (long chain triglycerides), petroleum distillates (including mineral oil), diluent terpenes, cannabinoids, and nicotine.

¶ https://www.jsonline.com/story/news/crime/2019/09/16/wisconsin-brothers-charged-huge-counterfeit-vaping-cartridge-bust/2346311001.
